# Full-length transcriptome sequences and the identification of putative genes for flavonoid biosynthesis in safflower

**DOI:** 10.1186/s12864-018-4946-9

**Published:** 2018-07-24

**Authors:** Jiang Chen, Xiaohui Tang, Chaoxiang Ren, Bin Wei, Yiyun Wu, Qinghua Wu, Jin Pei

**Affiliations:** 1 State Key Laboratory Breeding Base of Systematic Research, Development and Utilization of Chinese Medicine Resources, Chengdu, 611137 China; 2 College of Pharmacy, Chengdu University of Traditional Chinese Medicine, Chengdu, 611137 China; 3 College of Agronomy, Sichuan Agricultural University, Chengdu, 611130 China

**Keywords:** Full-length transcriptome, Flavonoid biosynthesis, Gene expression, MeJA treatment, PacBio RS II, Safflower

## Abstract

**Background:**

The flower of the safflower (*Carthamus tinctorius* L.) has been widely used in traditional Chinese medicine for the ability to improve cerebral blood flow. Flavonoids are the primary bioactive components in safflower, and their biosynthesis has attracted widespread interest. Previous studies mostly used second-generation sequencing platforms to survey the putative flavonoid biosynthesis genes. For a better understanding of transcription data and the putative genes involved in flavonoid biosynthesis in safflower, we carry our study.

**Results:**

High-quality RNA was extracted from six types of safflower tissue. The RNAs of different tissues were mixed equally and used for multiple size-fractionated libraries (1–2, 2–3 and 3-6 k) library construction. Five cells were carried (2 cells for 1–2 and for 2-3 k libraries and 1 cell for 3-6 k libraries). 10.43Gb clean data and 38,302 de-redundant sequences were captured. 44 unique isoforms were annotated as encoding enzymes involved in flavonoid biosynthesis. The full length flavonoid genes were characterized and their evolutional relationship and expressional pattern were analyzed. They can be divided into eight families, with a large differences in the tissue expression. The temporal expressions under MeJA treatment were also measured, 9 genes are significantly up-regulated and 2 genes are significantly down-regulated. The genes involved in flavonoid synthesis in safflower were predicted in our study. Besides, the SSR and lncRNA are also analyzed in our study.

**Conclusions:**

Full-length transcriptome sequences were used in our study. The genes involved in flavonoid synthesis in safflower were predicted in our study. Combined the determination of flavonoids, *CtC4H2*, *CtCHS3*, *CtCHI3*, *CtF3H3*, *CtF3H1* are mainly participated in MeJA promoting the synthesis of flavonoids. Our results also provide a valuable resource for further study on safflower.

**Electronic supplementary material:**

The online version of this article (10.1186/s12864-018-4946-9) contains supplementary material, which is available to authorized users.

## Background

The safflower, *Carthamus tinctorius* L., is a member of the family Asteraceae and is an important economic plant worldwide. As a traditional Chinese medicine, the dried flower of safflower has been widely used to improve cerebral blood flow and to treat coronary heart disease, hypertension, and cerebrovascular and gynaecological diseases [1, 2]. Flavonoids, particularly the water-soluble components, are responsible for these therapeutic effects. Among the flavonoids, hydroxysafflor yellow A (HYSA), the primary active component of safflor yellow, has antioxidation activities and myocardial and cerebral protective effects [3–6].

Currently, the basic metabolic pathway of flavonoid biosynthesis is more clearly defined, particularly in *Arabidopsis thaliana* [7–9]. However, the biosynthesis of flavonoids in safflower remains largely unknown. The identification of putative genes for flavonoid biosynthesis is highly significant not only for clarifying the accumulation of active ingredients of safflower but also for the application of biotechnology to improve their biosynthesis. To date, the focus of most of the published papers on safflower has been on the use of second-generation sequencing platforms to survey the putative genes and their metabolites. Huang et al. used Illumina-based de novo transcriptome sequencing to discover all known genes and primary metabolic pathways in this transcriptome [10] 156 unigenes as encoding enzymes involved in flavonoid synthesis were identified based on the KEGG pathway assignments. Liu et al. used 454 pyrosequencing to investigate genes related to the biosynthesis of safflor yellow [11], 22 unigenes, mainly including chalcone synthase genes, chalcone isomerase genes and anthocyanidin synthase genes were identified [11]. and Li et al. used Solexa-based deep sequencing to study oleosin-coding genes and to investigate genes related to flavonoid biosynthesis and metabolism in safflower [12]. Based on these sequence data, some genes involved in flavonoid biosynthesis in safflower have been cloned, such as UDP-glycosyltransferase [13] and flavanone 3-hydroxylase (F3H) [14]. Although some genes involved in flavonoid biosynthesis have been cloned, many problems are associated with gene cloning and metabolic analysis based on second-generation transcript data, such as the short read length, which is a huge drawback, and the tendency to error splicing, among others [15].

With the development of sequence technology, third-generation sequencing platforms can now be used to sequence full-length transcripts, which increase the accuracy of transcriptome characterisation compared with the transcript tags assembled from second-generation sequencing platforms. Among the third-generation sequencing platforms, PacBio RS II is the first commercialised third-generation DNA sequencer, which uses a novel and unique single molecule real-time (SMRT) technology [16]. PacBio RS II can provide much longer read length than the second-generation sequencing platforms, and this technology confers four primary advantages compared with other sequencing technologies: long read lengths, high consensus accuracy, a low degree of bias, and a simultaneous capability of epigenetic characterisation [17]. Therefore, the technology is widely used in genome and transcript sequencing [18–21]. However, except for several papers on *Salvia miltiorrhiza* [22, 23], few reports are on the use of third-generation sequencing with medicinal plants.

In this study, for a better understanding of transcription data, PacBio RS II was used to sequence the full-length transcriptome for safflower. All the genes involved in flavonoid biosynthesis were screened, and their expression patterns were analysed. Additionally, the temporal expression of genes under MeJA treatment was also measured. Our results not only provide a better understanding of flavonoid biosynthesis in safflower but also provide a valuable resource for further study in safflower.

## Methods

### Plant material

Safflower plants were cultivated in 2015 at the medicinal botanical garden, Wenjiang campus, Chengdu University of Traditional Chinese Medicine. Roots, stems and leaves and petals (the first, third and fifth days after anthesis (DAA) were collected. For each sample, tissues from at least 5 plants, which the genetic background and growth rate are consistent, were pooled. Samples were immediately frozen in liquid N_2_ for the RNA sequencing and expression analysis.

### RNA preparation

All the tissues were grinded on dry ice and the total RNA was prepared by TRIzol reagent (Invitrogen, CA, USA). To remove DNA, an aliquot of total RNA was treated with DNase (Takara, Dalian, China). The same amount of RNA from each sample (roots, stems, leaves and petals) was mixed for sequencing analysis. To ensure the accuracy of sequencing data, a Nanodrop was used to detect RNA purity (OD 260/280), concentration, and nucleic acid absorption peak, and an Agilent 2100 was used to detect RNA integrity, with detection indicators that included an RIN value, 28S/18S.

### PacBio Iso-Seq library preparation and sequencing

Total RNA (15 μg) was reversely transcribed into cDNA using a SMARTer™ PCR cDNA Synthesis Kit that was optimized for preparing high-quality, full-length cDNAs (Takara). BluePippin™ Size Selection System (Sage Science, Beverly, MA) was used to construct cDNA libraries of different sizes: 1–2, 2–3, and 3–6 k. Then, PCR was used to amplify the full-length cDNA, repair the end of full-length cDNA and connect the SMRT dumbbell-type connector. Blue Pippin was used for secondary screening to obtain the sequencing libraries. When the library size was consistent with the expected, sequencing could be performed. Sequencing was conducted on the Pacific Bioscience RS II platform using C3 reagents with 120 min movies. The raw data was upload to Sequence Read Archive (SRA) (http://www.ncbi.nlm.nih.gov/) with accession SRR6123576 (1–2 k data), SRR6123575 (2–3 k data) and SRR6123574 (3–6 k data).

### Analysis of the full-length Transcriptome

The analysis of the full-length transcriptome consisted of three stages [24]: full-length sequence recognition, isoform-level clustering to obtain a consistent sequence and a consistent sequence of polishing. First, the Reads Of Insert (ROI) sequences extracted from the original depot sequence had the cDNA primers and polyAs in the sequence filtered, and then the sequence was divided into sequences according to whether the 3′ primer, 5′ primer and polyA (optional), long and non-full-length sequences, and chimeric sequences and non-chimeric sequences were present. Then, the iterative isoform-clustering algorithm was used to cluster the full-length sequences from the same isoform, and the full-length sequences with similar sequences were clustered. A consistent sequence was contained in each cluster. Lastly, using the Quiver algorithm to cluster non-full-length sequences, the resulting consistent sequences were polished, and the high-quality sequences were screened for subsequent analysis. Considering the limitations of a cDNA library, we screened the high-quality sequences because the deletion of the 5 ‘end of a sequence in the library might indicate a non-full-length sequence: therefore, we only pooled 5’ exon sequences, and the longest sequence was used as the final transcript sequence.

### Phylogenetic analysis of flavonoid biosynthesis genes

Genes annotated to flavonoid biosynthesis were selected. Simultaneously, the functional genes that participate in flavonoid synthesis in rice and *Arabidopsis thaliana* were selected from NCBI and then pooled together before performing an alignment with MEGA 5.0 (MEGA, http://www.megasoftware.net/). We then constructed an phylogenetic tree using the neighbour-joining clustering method with the full-length amino acid sequences.

### Expression analysis of flavonoid biosynthesis gene families in safflower

In general, semi-quantitative PCR is used when the accuracy of gene expression is not high and the expression within differential treatments show a big difference. It was used more in tissue-specific expression analysis. Here, we analysed the expression of the annotated flavonoid biosynthesis genes using semi-quantitative RT-PCR. All gene specific primers were designed to amplify products of 100–400 bp in length. The length of the primers was 20 ± 2 bp. The specific primer for the 28S gene was used as the internal control. The specificity was tested by agarose gel electrophoresis. The detailed PCR primer sequences are shown in Additional file 1: Table S1. RNA was isolated from roots, stems, leaves, and three developmental stages of safflower. Total RNA was extracted using Trizol reagent (Invitrogen) according to the manufacturer’s instructions and was treated with DNase I (Takara). First-strand synthesis of cDNA was performed using RR047 kit (Takara) (1 μg of total RNA was used for cDNA synthesis in a 20 μl reaction volume). In order to adjust the PCR reaction for flavonoid biosynthesis gene, the amount of the template was adjusted, and then the semi-quantitative PCR reaction of the flavonoid biosynthesis gene was carried out. The expression of each flavonoid biosynthesis gene was detected by gel electrophoresis.

### Expression analysis of flavonoid biosynthesis gene families under MeJA treatment

The treatment was primarily applied according to a previous report with some modification [13]. A 100 μM solution of MeJA (Sigma-Aldrich) was sprayed onto healthy safflower flowers at 3 DAA. In the control group, the flowers were sprayed with the same solution but without MeJA. To minimize the errors possibly resulting from the differences between individual plants, five flowers were sprayed for each treatment, and each flower was consecutively sprayed five times. The flowers were then enclosed with clear plastic bags to prevent the emission of volatile phytohormones and allow the elicitor solutions to be more highly absorbed. After treatment for 6 h, the plastic bags were removed, and samples of flowers were collected, frozen immediately in liquid nitrogen and stored in a freezer at − 80 °C. The RNA for each sample was extracted as before. The primers are listed in Additional file 1: Table S1. we used quantitative RT-PCR to analysed the expression of the flavonoid biosynthesis genes under MeJA treatment. Quantitative RT-PCR analysis was conducted in triplicate using SYBR Premix Ex Taq TM II (TaKaRa), with 28S as a reference gene, on a Bio-Rad CFX96 system (Bio-Rad, CA, USA).

## Results

### Full-length sequencing and Acquisition of High-quality Redundant Sequences

**Fig. 1:**
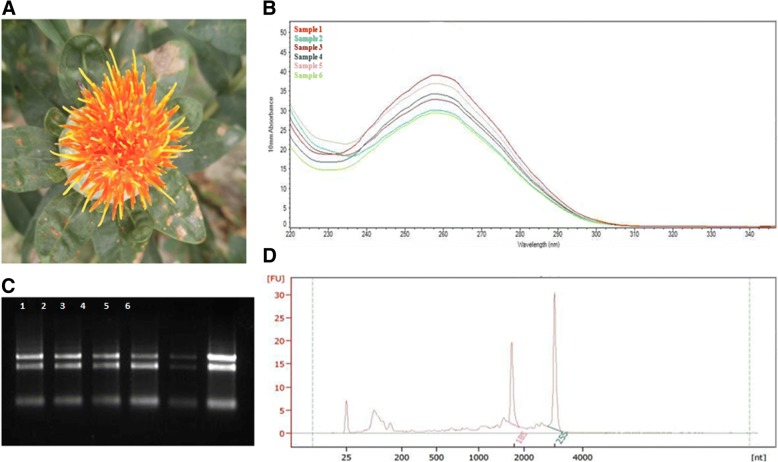
Extraction and validation of high quality RNA. **a** The safflower at third day after anthesis (DAA). **b** Nanodrop map of the RNA. Sample 1 to 6 represent the RNA of different tissues (roots, stems, leaves, and petals 1, 3 and 5 DAA). **c** RNA gel electrophoresis. 1–6 represent the RNA of different tissues (roots, stems, leaves, and petals 1, 3 and 5 DAA). D: RNA integrity detected by agilent 2100. The 18 s and 28 s was marked in the figure

To identify as many transcripts as possible, high-quality RNA was extracted from six types of safflower tissue (roots, stems, leaves, seeds, and petals 1 and 3 DAA) (Fig. 1). In order to get more RNA sequences, which can represent the gene expression of the whole plant, the RNAs of different tissues were mixed equally and then used for library construction. Multiple size-fractionated libraries (1–2, 2–3 and 3-6 k) were constructed to avoid loading bias, which favours sequencing of short transcripts. Five cells were carried (2 cells for 1–2 and for 2-3 k libraries and 1 cell for 3-6 k libraries), yielding 517,898 reads. Filtered for subread length less than 50 bp and sequence accuracy less than 0.75, a total of 4,548,120 subreads (10.43Gb of clean data) were obtained (Additional file 1: Table S2). Each size-selected library had the expected distribution of transcript lengths, ranging from 500 to 4900 bp (Additional file 2: Figure S1). Of the reads of insert, 196,114 of 338,902 were full-length reads based on the presence of bar-coded primers and polyA tails (Additional file 1: Table S3).

The SMRT Analysis (v2.3.0) software using the ICE (Iterative Clustering for Error Correction) algorithm, combined with the quiver program, was used for sequence clustering, and a total of 79,926 isoforms were obtained of which HQ (High-Quality) transcripts were 60,894 and LQ (Low-Quality) transcripts were 19,032 (Additional file 1: Table S4). Because a draft genome assembly of safflower (two-thirds of the expected genome) has been reported [25], two methods were used with the HQ transcripts to obtain the de-redundant data. In one method, HQ transcript sequences were mapped to the draft genome using GMAP (Genomic Mapping and Alignment Program) [26]. The sequences with identity less than 0.9 and coverage less than 0.85 were filtered. Reads differing only at the 5′-start site within the first exon were counted as redundant reads. Using this method, 19,352 non-redundant transcript sequences were obtained. With the other method, because the HQ transcript sequences were not fully matched with the draft genome, we used CD-HIT to remove redundant sequences from high-quality transcripts according to sequence similarity [27]. The sequences with similarity of 0.95 were clustered and resulted in a sequence of 18,950 transcripts. Ultimately, a total of 38,302 de-redundant sequences were obtained. The workflow for data processing is listed in Additional file 3: Figure S2.

### Functional annotation

The set of 38,302 unique isoforms was annotated using BLASTX (version 2.2.26) and a variety of protein databases (COG (Clusters of Orthologous Groups) [28], GO (Gene Ontology) [29], KEGG (Kyoto Encyclopedia of Genes and Genomes) [30], KOG (euKaryotic Ortholog Groups) [31], Pfam (Protein family) [32], Swissprot [33], NR [34]. The new isoforms were annotated, and the details referred to the Supplementary (Additional file 1: Table S5).

**Fig. 2:**
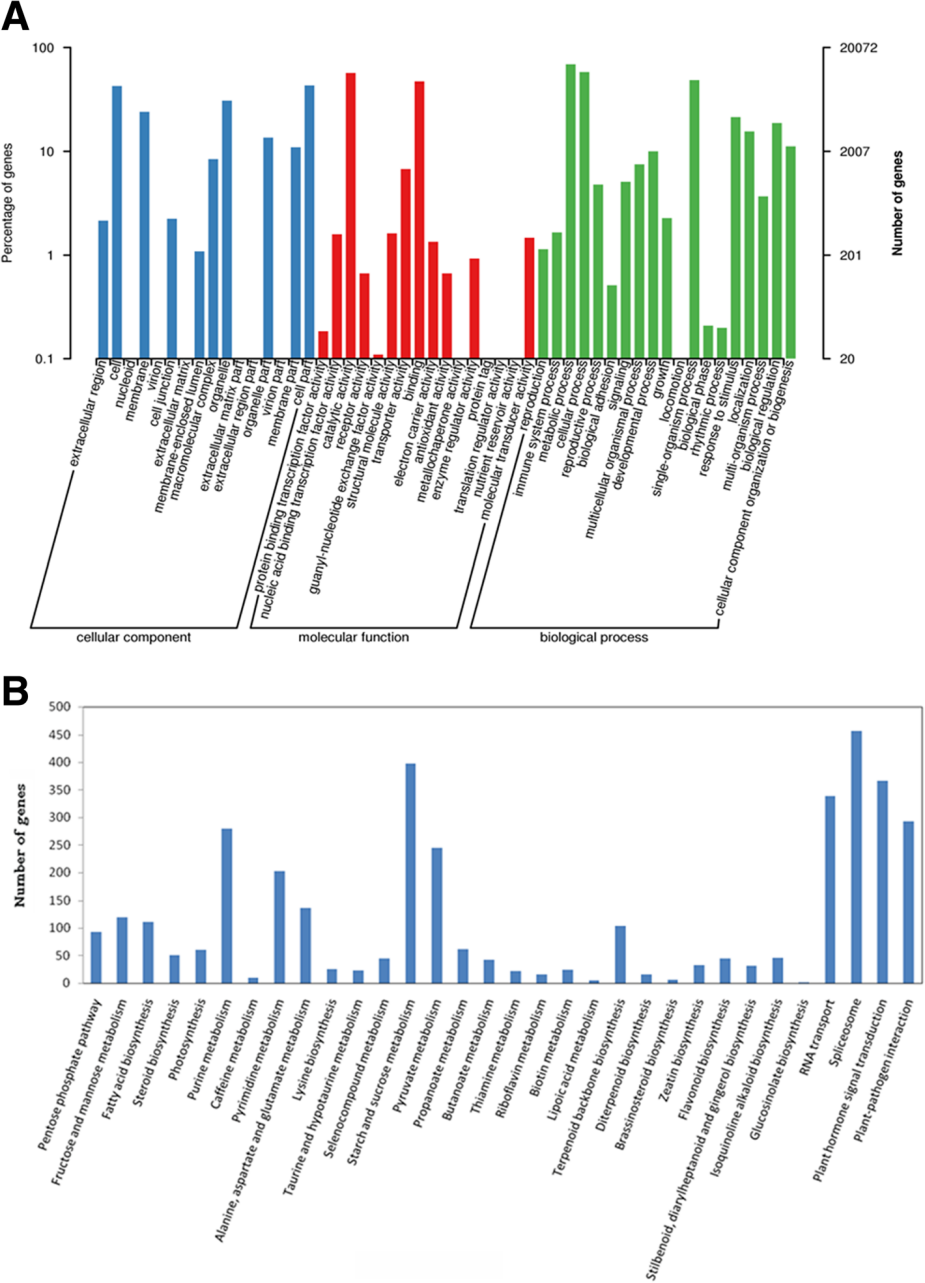
GO and KEGG annotation of the safflower de-redundant sequences. **a** GO annotation of all the safflower de-redundant sequences. Three primary GO categories and 51 subcategories (functional groups) were summarised into GO. **b** KEGG annotation of the safflower de-redundant sequences. Only some of the significant pathways was listed in the figure

GO functional annotations from these databases were used to assign molecular function, cellular component, and biology process terms to the safflower unique isoforms. Three primary GO categories and 51 subcategories (functional groups) were summarised into GO. A high percentage of the genes fell under “metabolic process”, “cellular process” and “single-organism process” of the biological processes category; “binding” and “catalytic activity” dominated in the molecular function category; and a high percentage of the genes fell under “cells,” “cell parts,” and “organelles” of the cellular components category (Fig. 2a).

For the unigenes, biological pathways, including metabolic, signal transduction, and genetic information processing pathways, were identified by KEGG pathway analysis. A total of 126 pathways were evaluated (Additional file 1: Table S6), with some of the significant pathways listed in Fig. 2b. Significant pathways containing many isoforms were for plant hormone signal transduction, starch and sucrose metabolism, spliceosome, RNA transport, and plant-pathogen interaction.

### Candidate genes involved in flavonoid biosynthesis

**Fig. 3:**
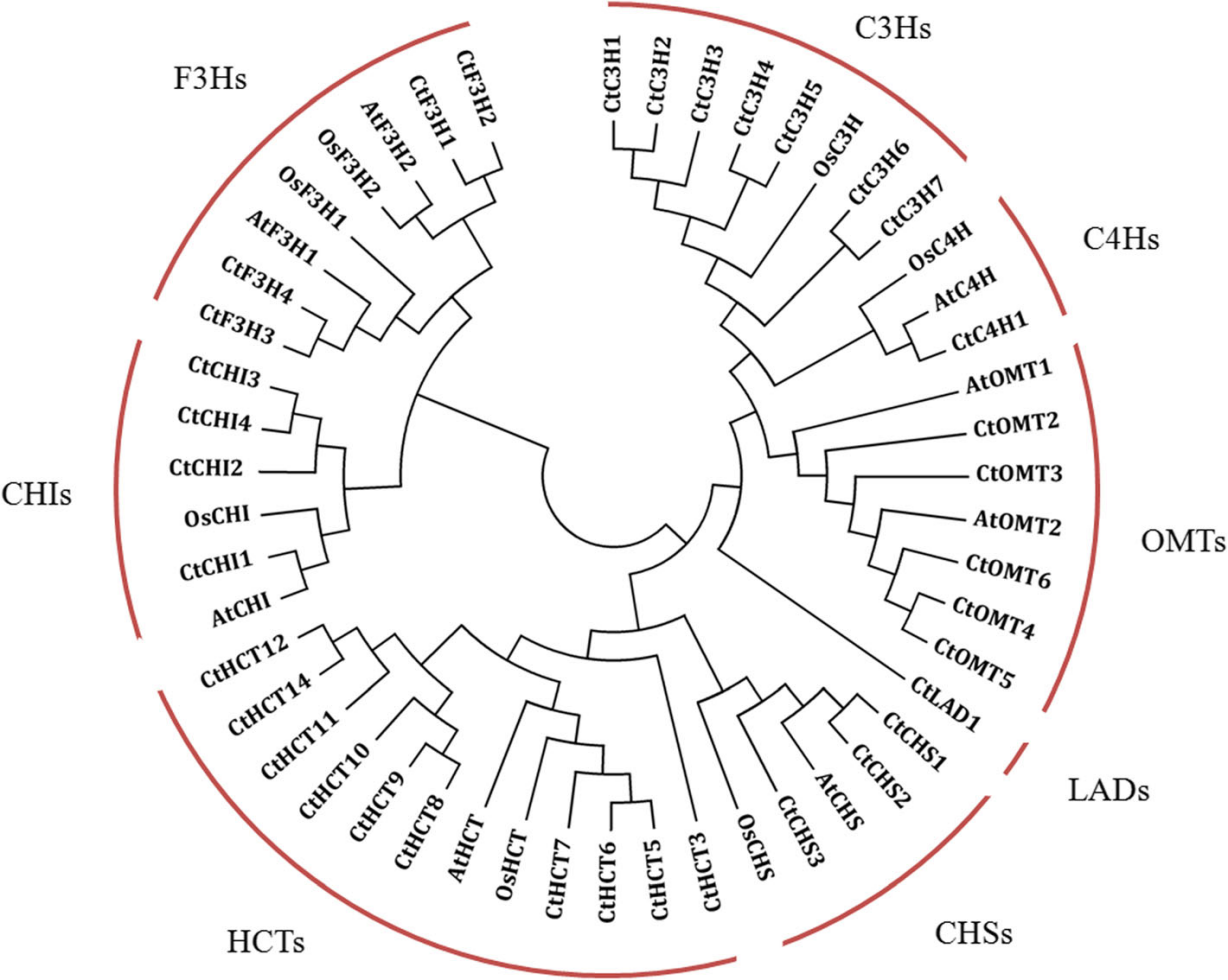
Phylogenetic relationships of flavonoid synthesis genes with safflower, rice and *Arabidopsis thaliana*. The phylogenetic tree was constructed using MEGA 5.0 with the neighbor-joining method. The safflower flavonoid genes were divided into 8 families: C3H, C4H, OMT, LAD, HCT, CHI, CHS and F3H. According to the clustering results, we named the flavonoid biosynthesis genes in safflower

Flavonoids are the primary bioactive components in safflower, and their biosynthesis has attracted widespread interest. In this study, we focused on the genes involved in flavonoid biosynthesis. According to the KEGG pathway assignments, 44 unique isoforms were annotated as encoding enzymes involved in flavonoid biosynthesis. The flavonoid biosynthesis genes information was listed in Additional file 1: Table S7. Because flavonoid synthesis is more clearly defined in some model plants (*Arabidopsis thaliana* and rice, among others), the flavonoid biosynthesis genes in rice and *Arabidopsis* (Additional file 1: Table S8) were used to blast with the annotated flavonoid biosynthesis genes in safflower. According to the clustering results (Fig. 3), we named the flavonoid biosynthesis genes in safflower. The safflower flavonoid genes were divided into 8 families: C3H, C4H, OMT, LAD, HCT, CHI, CHS and F3H.

**Fig. 4:**
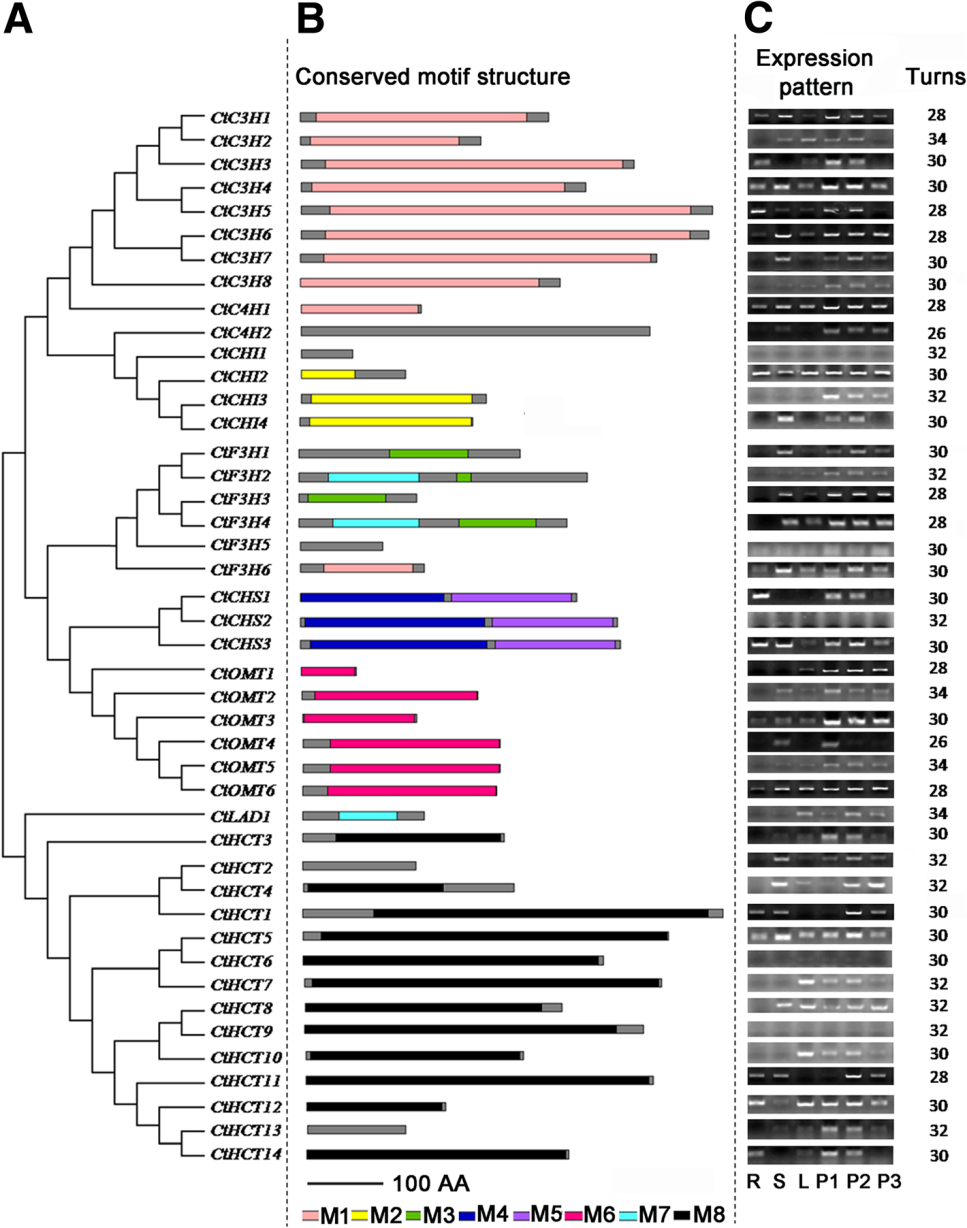
The structure and expression analyses of flavonoid biosynthesis genes. **a** The phylogenetic relationships of flavonoid syntheses genes in safflower. The phylogenetic tree was construct by MEGA 5.0. **b** The conserved motif structure analyses. The protein sequences were analysis on Pfam (http://pfam.xfam.org/). M1 represents Cytochrome P450 domain, M2 represents Chalcone-flavanone isomerise domain, M3 represents 2OG-Fe(II) oxygenase superfamily domain, M4 represents Chalcone and stilbene synthases domain, M5 represents N-terminal domain, M6 represents O-methyltransferase domain, M7 represents non-haem dioxygenase in morphine synthesis N-terminal domain, M8 represents transferase family domain. **c** Expression analyses by semi-quantitative RT-PCR. R represents roots, S represents stems, L represents leaves, P1–3 represents petals at the first, third and fifth days after anthesis (DAA)

To further define the annotation of the flavonoid biosynthesis genes, their conserved motif structure was analysed (Fig. 4b). Both C3H and CtC4H families had a cytochrome P450 domain, and this domain iscomposed of haem-thiolate proteins involved in the oxidative degradation of various compounds. These families are involved in a wide range of biosynthetic reactions that lead to the biosynthesis of plant hormones, secondary metabolites and lignins, among others [35]. The CHI family contained thechalcone-flavanone isomerase domain. Chalcone-flavanone isomerase is a plant enzyme responsible for the isomerisation of chalcone to naringenin, 4′,5,7-trihydroxyflavanone, a key step in the biosynthesis of flavonoids [36]. The F3H family had two domains. One domain was the 2OG-Fe(II) oxygenase superfamily, which contains members of the 2-oxoglutarate (2OG) and Fe(II)-dependent oxygenase superfamilies [37]. The full enzyme consists of an alpha2 beta2 complex with the alpha subunit contributing most parts of the active site [38]. The non-haem dioxygenase in morphine synthesis N-terminal was the other domain, which is the highly conserved N-terminal region of proteins with 2-oxoglutarate/Fe(II)-dependent dioxygenase activity [39]. The family of CHS contained the C-terminal domain. CHS is an important enzyme in flavonoid biosynthesis, catalysing the addition of three molecules of malonyl-CoA to a starter CoA ester (a typical example is 4-coumaroyl-CoA), producing a chalcone. The OMT family contained an O-methyltransferase domain. The domain includes catechol O-methyltransferase and caffeoyl-CoA O-methyltransferase and a family of bacterial O-methyltransferases that may be involved in antibiotic production [40]. The OMT enzyme shows a strong preference for methylating the para position of flavanones and dihydroflavonols, whereas flavones and flavonols are methylated in the meta-position [41] . It was predicted to involve in the process of Caffeoyl-CoA to Feruloyl-CoA conversion in flavonoid biosynthesis. The CtHCT family contains several transferase enzymes, which include anthranilate N-hydroxycinnamoyl/benzoyltransferase that catalyses the first committed reaction of phytoalexin biosynthesis [42] and deacetylvindoline 4-O-acetyltransferase EC:2.3.1.107 that catalyses the last step in vindoline biosynthesis [43].

### Expression analyses of candidate flavonoid biosynthesis genes

To further analyse flavonoid biosynthesis genes in safflower, we performed semi-quantitative PCR (semi-qPCR) analysis of their expression patterns (Fig. 4c). Based on the results, the expression of flavonoid biosynthesis genes showed large differences. In the C3H family, the expressions of *CtC3H2*, *CtC3H7*, and *CtC3H8* were low compared with that of *CtC3H4*, *CtC3H5* and *CtC3H6*. In the CHI family, the expression of *CtCHI4* was lower than that of *CtCHI2*, and in the F3H family, the expressions of *CtF3H2* and *CtF3H6* were low compared with that of *CtF3H3* and *CtF3H4*.For the CHS family, the expression of *CtCHS3* was relatively higher than that of *CtCHS1*, and in the OMT family, the expression of *CtOMT2* and *CtOMT5* was low compared with that of *CtOMT3* and *CtOMT6*. In the HCT family, the expression of *CtHCT2*, *CtHCT7*, *CtHCT8*, and *CtHCT13* was low compared with that of *CtHCT5*, *CtHCT10*, and *CtHCT11*.

For the specific expression in safflower organs, *CtC3H4*, *CtC3H5* and *CtC3H6* were expressed in various parts (roots, stems, leaves and flowers), whereas others had limited tissue-expression, such as *CtC3H2*, which was not expressed in the roots, and *CtC3H3*, which was not expressed in the stems. In contrast to the expression of *CtCHI2*, *CtCHI3* and *CtCHI4* had restricted tissue expression, with *CtCHI3* only expressed in flowers and *CtCHI4* without expression in roots and leaves. Most *CtF3Hs* were not expressed in roots, whereas *CtCHS1* was primarily expressed in flowers. *CtOMT3* and *CtOMT6* were expressed in various parts(roots, stems, leaves and flowers); whereas *CtOMT2* and *CtOMT4* had restricted tissue expression. In contrast to the expression of *CtHCT5*, most *CtHCTs* had restricted tissue expression, with no expression in roots.

Because the ingredients in safflower, particularly medicinally active ingredients, are primarily expressed in flowers, we assumed that the genes that were primarily expressed and had high expression in flowers were more likely to be involved in flavonoid biosynthesis in safflower than other genes, which included *CtC4H1*, *CtC4H2*, *CtC3H1*, *CtC3H5*, *CtC3H6*, *CtCHI2*, *CtCHI3*, *CtF3H1*, *CtF3H3*, *CtF3H4*, *CtCHS1* and *CtCHS3*, among others. By contrast, the other genes that had little expression in flowers, such as *CtCHI1*, *CtF3H5*, *CtCHS2*, *CtHCT6* and *CtHCT9*, were likely not involved in flavonoid biosynthesis in flowers.

### Temporal expression analyses of flavonoid biosynthesis genes under MeJA treatment

**Fig. 5:**
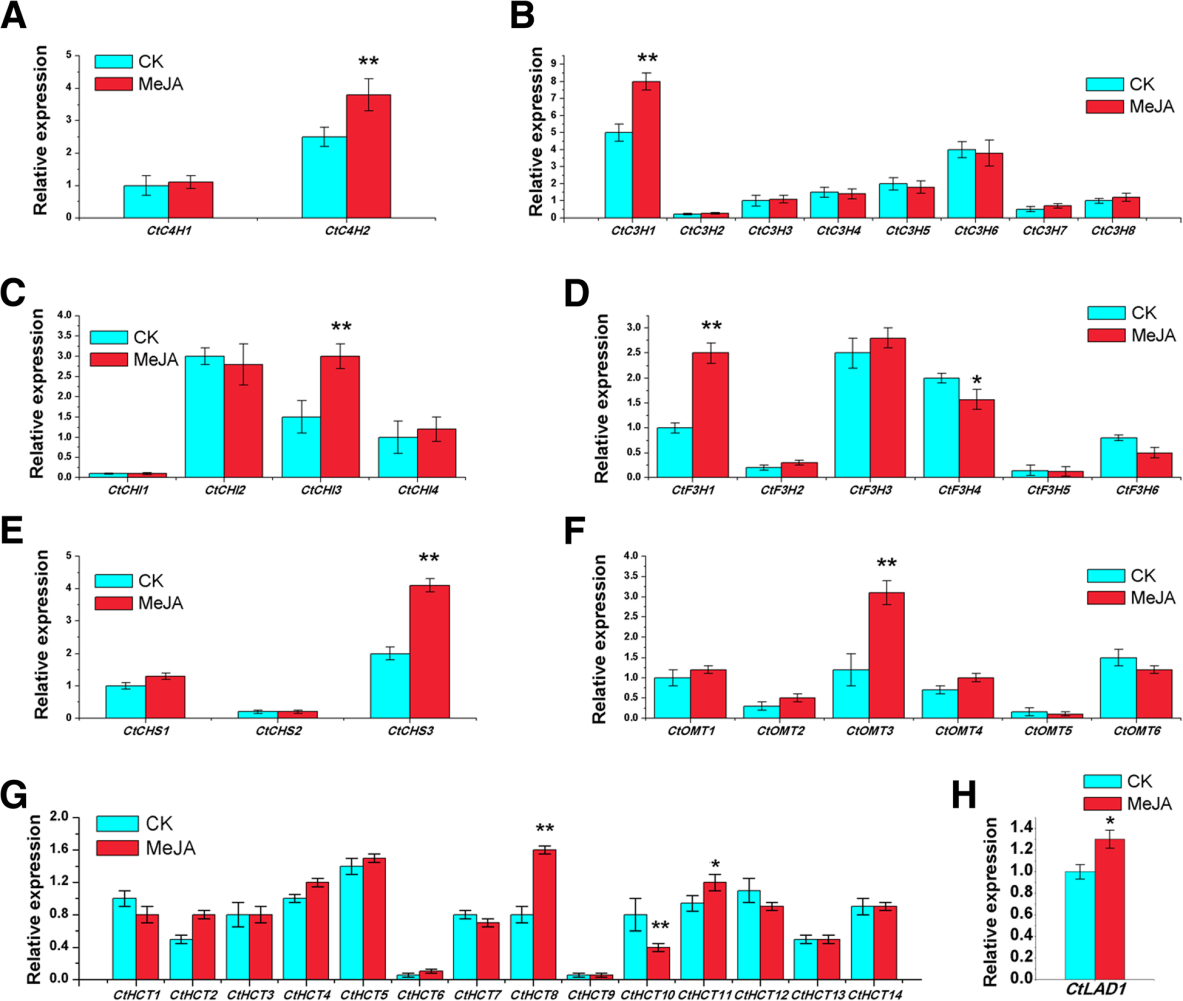
Temporal expression analyses of flavonoid biosynthesis genes under MeJA treatment. **a** Temporal expression of *CtC4Hs* under MeJA treatment. **b** Temporal expression of *CtC3Hs* under MeJA treatment. **c** Temporal expression of *CtCHIs* under MeJA treatment. **d** Temporal expression of *CtF3Hs* under MeJA treatment. **e** Temporal expression of *CtCHSs* under MeJA treatment. **f** Temporal expression of *CtOMTs* under MeJA treatment. **g** Temporal expression of *CtHCTs* under MeJA treatment. **a** Temporal expression of *CtLAD* under MeJA treatment. The significance of the difference was analyzed using a one-sided paired t-test (* *P* < 0.05, ** *P* < 0.01)

As a well-known exogenous inducing factor, MeJA participates in many plant processes, ranging from plant defence to growth and development [44]. MeJA is of particular interest in plant cell engineering for producing bioactive compounds [45, 46] and most flavonoids in safflower (safflor yellow A, naringenin, dihydro-kaempferol, kaempferol, quercetin) are stimulated under MeJA treatment [13]. To investigate the response of flavonoid biosynthesis pathways to MeJA, the expression patterns of the flavonoid biosynthesis genes were detected under MeJA treatment (Fig. 5).

In the C4H family, MeJA significantly increased *CtC4H2* gene expression. In the C3H family, MeJA has a significant up-regulation of *CtC3H1*, but little effect on other genes. In the CHI family, MeJA has a significant up-regulation of *CtCHI3*, but little effect on other genes. In the F3H family, MeJA has a significant up-regulation of *CtF3H1* and a significant down-regulation of *CtF3H4*. In the CHS family, MeJA has a significant up-regulation of *CtCHS3*. In the OMT family, MeJA has a significant up-regulation of *CtOMT3*, but little effect on other genes. In the HCT family, MeJA has a significant up-regulation of *CtHCT8* and a significant down-regulation of *CtHCT11.* Besides, MeJA has a significant up-regulation of *CtLAD1* All in all, 9 genes are significantly up-regulated, including *CtC4H2*, *CtC3H1*, *CtCHI3*, *CtF3H4*, *CtCHS3*, *CtOMT3*, *CtHCT8*, *CtHCT11* and *CtLAD1*, and 2 genes are significantly down-regulated, especially *CtF3H4* and *CtHCT10*.

### The analysis of SSR and lncRNA

In this study, we mainly used full-length analysis for the identification of flavonoid synthesis functional genes. We also analysed SSR and lncRNA. SSRs have been a common source of markers for genetic mapping, molecular breeding, and population genetic analyses in a wide variety of species [47]. MISA software was used for SSR analysis, and six types of SSR were identified: mono-nucleotide, di-nucleotide, tri-nucleotide, tetra-nucleotide, penta-nucleotide, andhexa-nucleotide; for details, see Additional file 1: Table S9. LncRNAs are non-coding RNA transcripts bigger than 200 nucleotides in length and pervasively expressed across the genome. Disruption and mis-expression of lncRNAs has been shown to be associated with malfunctioning in an organism development and observed phenotypic differences in plants [48]. As LncRNA does not encode a protein, to determine whether the transcript was lncRNA, transcripts were screened for coding potential. The most widely used current coding potential analysis methods were used, including CPC [49], CNCI [50], pfam [32], and CPAT [51]. For the visual analysis of the results, the noncoding transcripts identified by these four types of analysis software are shown in a Venn diagram(Additional file 4: Figure S3). The ORF prediction of the candidate lncRNAs predicted by the four methods was performed using EMBOSS, and the sequences of ORF length greater than 100 amino acids were filtered as the final lncRNA sequences. Ultimately, a total of 1247 lncRNA sequences were obtained from the 38,302 sequences (Additional file 1: Table S10).

## Discussion

SMRT data were of high quality in the study. First, high-quality RNA was obtained. The RIN was between 1.8 and 2.0, the 28S/18S was approximately 2.0, and OD 260/280 was approximately 2.0. Second, the length of the Reads Of Inserts was sufficient. The full-length transcripts had an average length of 1393, 2368 and 3729 bp for the 1–2, 2–3, and 3–6 k libraries, respectively. The ROI sequence was extracted from the original sequence according to the conditions of full passes greater than or equal to 0 and sequence accuracy greater than 0.75. In this sequence, the number of ROI sequences was 338,902 for which the sequence average mass of the 1-2 k cDNA library reached 0.94 and that of the 3-6 k cDNA library reached 0.92; the average mass value was 0.91. The values were 13, 8, and 7 for the sequencing depth (Additional file 1: Table S11). The full-length, non-chimeric reads were 196,114 (82,287 for 1-2 k, 74,012 for 2-3 k and 39,815 for 3-6 k). Artificial concatemers ratio was 0.22% (< 1%), and the data SMRT bell indicated moderate concentration (Additional file 1: Table S3). Third, many unique isoforms or alternative splicing events were specifically identified by SMRT derived reads. The longest transcripts of each gene in the upper genome were extracted as the reference transcript sequence, and cuffcomapare software was used to compare the variable splice sequence of this project with the reference transcript sequence (gff), using ASTALAVISTA software to analyse the variable splicing event (Additional file 5: Figure S4).

We obtained high quality de-hyperbolicism. A total of 38,302 full-length non-redundant sequences were obtained. We used two methods of data annotation and redundancy analysis. In addition to using GAMP to map transcript sequences to the genome, we used CD-HIT for de-redundancy. A sequence was considered a sequence with approximately 99% of the sequences. Of the 36,030 transcripts that were clustered, 22,616 transcripts were obtained. From the results of the return, the redundancy was not complete, with F3H as an example (Additional file 6: Figure S5).Thus we adjusted the parameters that a sequence was considered a sequence with approximately 95% of the sequences. The results from the 22,616 modulation sequences were further filtered to obtain 18,950 sequences. As shown in the results, the redundancy effect improved significantly. Besides, in order to determine the accuracy of the third-sequencing results, two genes (*CtCHS1* and *CtCHS3*) were randomly cloned and sequenced using the first-generations sequencing technology-Sanger method. Results show that the sequencing results were similar to the third-generations data, with 100% (*CtCHS1*) and 99% (*CtCHS3*), respectively (Additional file 7: Figure S6).

Huang et al. identified 156 unigenes as encoding enzymes involved in flavonoid synthesis based on the KEGG pathway assignments [10]. Liu et al. identified 22 unigenes, mainly including chalcone synthase genes, chalcone isomerase genes and anthocyanidin synthase genes [11]. We identified a total of 44 flavonoid genes. Although the flavonoid genes number identified in our study were far less than the second-generation data, but our data quality is higher than the previous data. The average length in reports of Huang et al. is about 748 bp (seed 692 bp; leaf 840 bp; petal 713 bp), while the average length in reports of Li et al. was 430 bp (early 427 bp; full 436 bp). In our data, the average length of our data is 2500 bp (1-2 k 1440 bp; 2-3 k 2426 bp; 3-6 k 3796 bp). We compared the flavonoid iosynthesis gene indentified in the previous study with our study. Most of the flavonoid biosynthesis genes were similar with that identified in our study. Such as in research of Huang et al., unigene34109, unigene46610, and unigene53415 were annotated as chalcone synthase [10]. We analysis the three genes with our data, results show that unigene34109 was similar to *CtCHS1*, unigene46610 was similar to *CtCHS2*, and *CtCHS3* identified as a new isoform. (Additional file 8: Figure S7A). Further analysis revealed that the reported *CHSs* were only part of our cloned gene sequence, such as unigene34109 (Additional file 8: Figure S7B). Besides, The full length of flavanone 3-hydroxylase gene had been cloned [14]. We also analysis the reported flavanone 3-hydroxylase gene with our data. Results show that the report *CtF3H* was similar to our data (*CtF3H2*) (Additional file 8: Figure S7C).

**Fig. 6:**
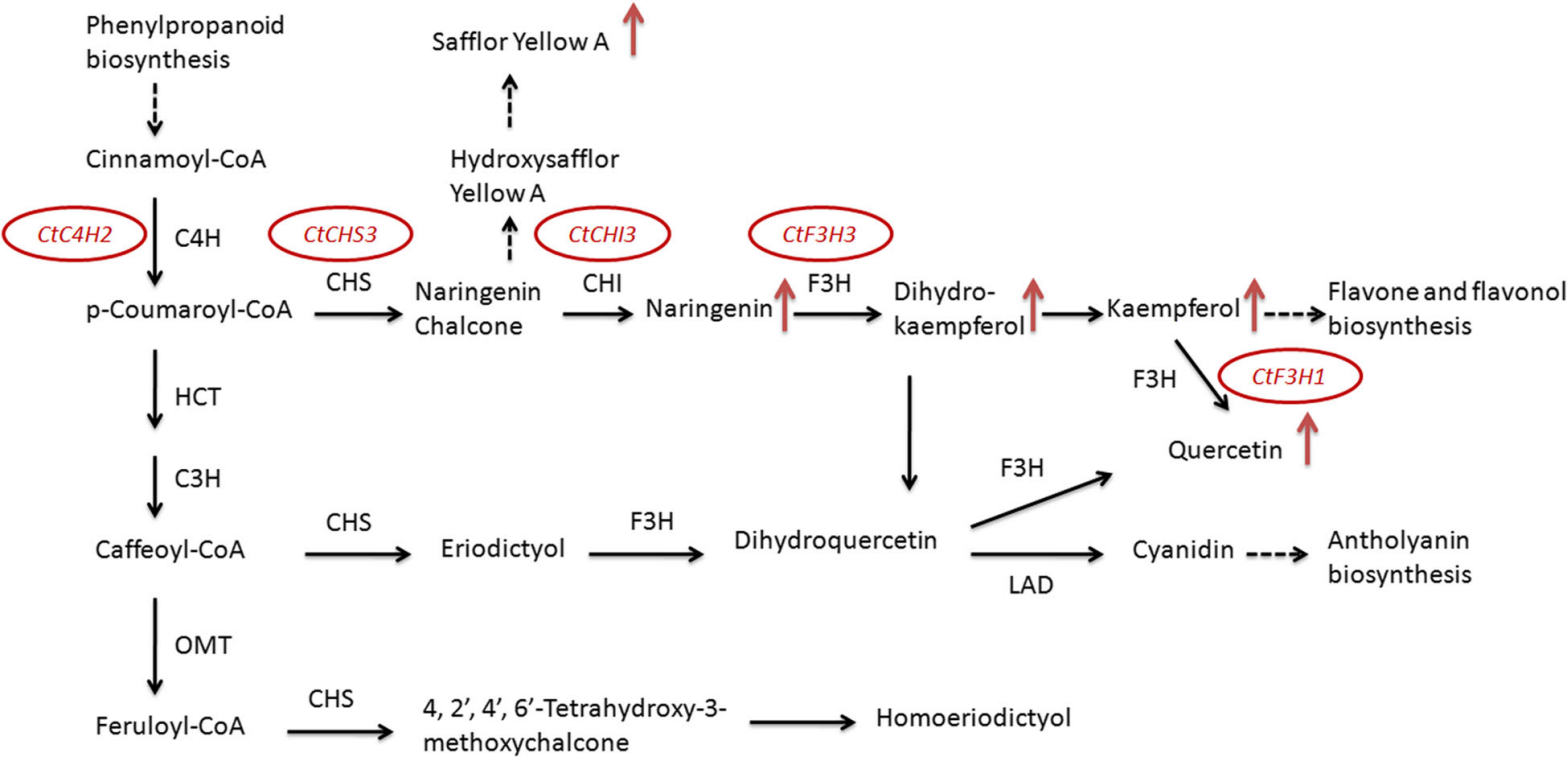
Flavonoid metabolic pathway in safflower and the genes significantly respond to MeJA treatment. The diagram was drawn conbined with the chemical composition and gene expression (including the tissue expression and the expression under MeJA treatment). Red arrow represents MeJA induces the content of this flavonoid. The genes inside the red circle were that significantly respond to MeJA treatment based on the gene expression analyses

The assumption was that the genes that were primarily expressed and had high expression in flowers were more likely to be involved in the biosynthesis of safflower flavonoids, which included *CtC4H1*, *CtC4H2*, *CtC3H1*, *CtC3H5*, *CtC3H6*, *CtCHI2*, *CtCHI3*, *CtF3H1*, *CtF3H3*, *CtF3H4*, *CtCHS1*, and *CtCHS3*, among others. Therefore, the other genes that had little expression in flowers, such as *CtCHI1*, *CtF3H5*, *CtCHS2*, *CtHCT6* and *CtHCT9*, were less likely to be involved in the biosynthesis of flavonoids in flowers. Combined with the flavonoids biosynthesis pathway in KEGG, we predicted the genes involved in flavonoid synthesis (Fig. 6). However, which isoform of each family involved in the specific metabolic needs further research. And it is possible to determine it by protein purification and activity assays of each isoform.

Based on the research of Guo et al. MeJA induces the production of flavonoids, including safflor yellow A, naringenin, dihydro-kaempferol, kaempferol and quercetin, We also measured the expression of flavonoid genes under MeJA treatment. 9 genes are significantly up-regulated, including *CtC4H2*, *CtC3H1*, *CtCHI3*, *CtF3H4*, *CtCHS3*, *CtOMT3*, *CtHCT8*, *CtHCT11* and *CtLAD1.* Combined with the flavonoids biosynthesis pathway we predicted, it is possible that MeJA promotes the synthesis of flavonoids through the 5 genes (*CtC4H2*, *CtCHS3*, *CtCHI3*, *CtF3H3*, *CtF3H1*) (Fig. 6). Although *CtC3H1*, *CtHCT8*, *CtHCT11* and *CtOMT3* also significant up-regulated, but from the results of Guo et al., the product of this metabolic pathway did not increase. There may be post-transcriptional regulation of these genes, which led to an increase in the amount of expression, but no increase in enzyme activity. Besides, 2 genes are significantly down-regulated, especially *HCT10*. The expression of *HCT8* and *HCT10* were just the opposite. We analysed the two isoforms and found that this gene showed an alternative 5′ splice site (Additional file 9: Figure S8). Because the external environment can change the alternative splicing [52], we predicted that the MeJA changed the type of AS.

## Conclusion

PacBio RS II was used to sequence the full-length transcriptome for safflower. Clean data, 10.43Gb, were obtained and 38,302 de-redundant sequences were captured. We screened all genes involved in the biosynthesis of flavonoids and analysed their expression patterns. Forty-four genes were divided into eight families that were annotated for involvement in the biosynthesis of flavonoids, and these genes showed large differences in expression. The genes involved in flavonoid synthesis in safflower were predicted in our study. The temporal expression of these genes under MeJA treatment was also measured. 9 genes are significantly up-regulated and 2 genes are significantly down-regulated. 5 genes are mainly participated in MeJA promoting the synthesis of flavonoids. Besides, the SSR and lncRNA are also analyzed in our study. Our results also provide a valuable resource for further study on safflower.

## Additional files

Additional file 1: **Table S1.** Primers used in RT-PCR. The gene name in our study, forword and reverse primer sequences are listed in the table. **Table S2.** Sequencing data statistics. The cDNA size, SMRT cells, post-Filter polymerase reads, and post-Filter Subreads N50, et al. are listed in the table. **Table S3.** Full length sequence data. The number of filtered short reads, number of non-full-length reads, number of full-length reads, et al. are listed in the table. **Table S4.** Clustering results by ICE. The average consensus isoforms read length, number of polished high-quality isoforms, et al. are listed in the table. **Table S5.** New Isoform predictions. **Table S6.** KEGG analyses. **Table S7.** The flavonoid biosynthesis gene information. The ID of flavonoid biosynthesis gene, the domain by pfam, et al. are listed in the table. **Table S8.** The flavonoid gene sequence in rice, *Arabidopsis thaliana*. The ID in NCBI, protein sequence, et al. are listed in the table. **Table S9.** SSR analysis. **Table S10.** lncRNA sequences ID. **Table S11.** ROI statistics. Reads of insert statistics. The cDNA size, the reads of insert, et al. are listed in the table. (XLSX 58 kb)

Additional file 2: **Figure S1.** The transcript lengths of each size-selected library. Each size-selected library had the expected distribution of transcript lengths, ranging from 500 to 4900 bp. (TIF 362 kb)

Additional file 3: **Figure S2.** The workflow for data processing in our study. (TIF 655 kb)

Additional file 4: **Figure S3.** Venn diagram of protein domain prediction. Four types of analysis software (cnci, cpc, pfam and cpat) were used. The intersection of the four software was used for the further lncRNA analyses. (PNG 670 kb)

Additional file 5: **Figure S4.** Variable splicing event analyses. The longest transcripts of each gene in the upper genome were extracted as the reference transcript sequence, and cuffcomapare software was used to compare the variable splice sequence of this project with the reference transcript sequence (gff), using ASTALAVISTA software to analyse the variable splicing event. (TIF 231 kb)

Additional file 6: **Figure S5.** Multiple sequence alignment of *F3Hs* after the first de-redundancy by CD-HIT. A sequence was considered a sequence with approximately 99% of the sequences. From the results, the three sequences are basically the same, which indicated that the redundancy was not complete. (TIF 1567 kb)

Additional file 7: **Figure S6.** Sequence alignment analysis between the first-generations data and the third-generations data using *CtCHS1* and *CtCHS3* as the examples. The sequencing results were similar to the third-generations data, with 100% (*CtCHS1*) and 99% (*CtCHS3*), respectively (TIF 3997 kb)

Additional file 8: **Figure S7.** Comparison with reported two generations data. A: The evolutionary analysis of CHS. B: Sequence alignment analysis between *CtCHS1* and UNIGENE34109. C: Sequence alignment analysis between *CtF3H2* and *CtF3H*. (TIF 3564 kb)

Additional file 9: **Figure S8.** Alternative splice analyses of *HCT8* and *HCT10*. A: Common sequence that *HCT8* and *HCT10* shared. B: The diagram of the alternative splice of *HCT8* and *HCT10*. (TIFF 2660 kb)

## Abbreviations

CHI: Chalcone isomerise
CHS: Chalcone synthase
COG: Clusters of Orthologous Groups
DAA: Days after anthesis
F3H: Flavanone 3-hydroxylase
GMAP: Genomic Mapping and Alignment Program
GO: Gene Ontology
HQ: High-Quality
HYSA: Hydroxysafflor yellow A
ICE: Isoform-clustering
ICE: Iterative Clustering for Error Correction
KOG: Karyotic Ortholog Groups
LncRNA: Long non-coding RNA
LQ: Low-Quality
Pfam: Protein family
semi-Qpcr: Semi-quantitative PCR
SMRT: Single molecule real-time
SRA: Sequence Read Archive
SSR: Simple sequence repeats
