# Supplementary figures and images for: Full-length transcriptome sequences and the identification of putative genes for flavonoid biosynthesis in safflower
Source: BMC Genomics. 2018 Jul 24;19:548. doi: 10.1186/s12864-018-4946-9 (PMC6057038; doi:10.1186/s12864-018-4946-9)

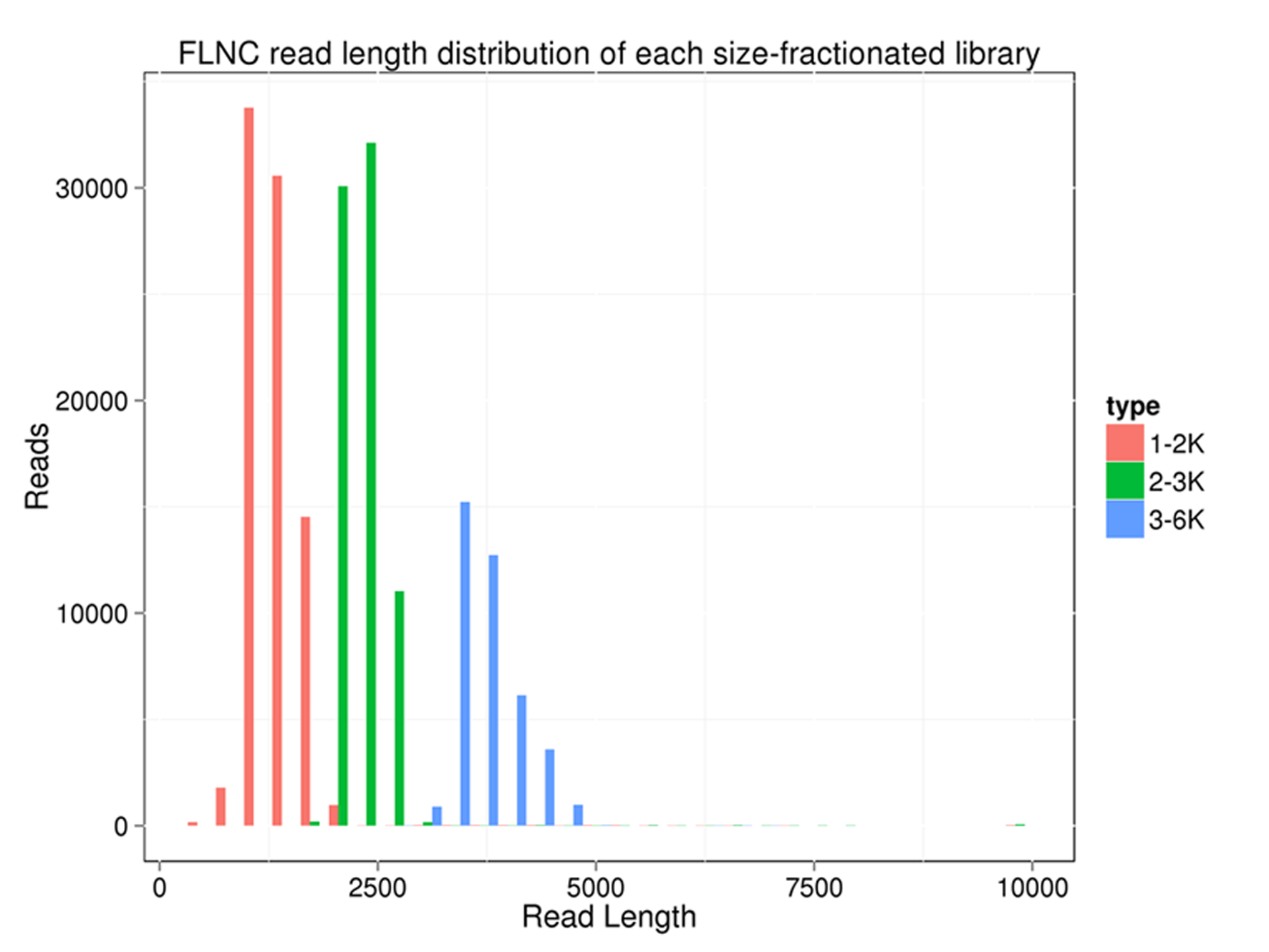

Supplement: Supplementary file 2 — Figure S1. The transcript lengths of each size-selected library. Each size-selected library had the expected distribution of transcript lengths, ranging from 500 to 4900 bp. (TIF 362 kb) [file 12864_2018_4946_MOESM2_ESM.tif]

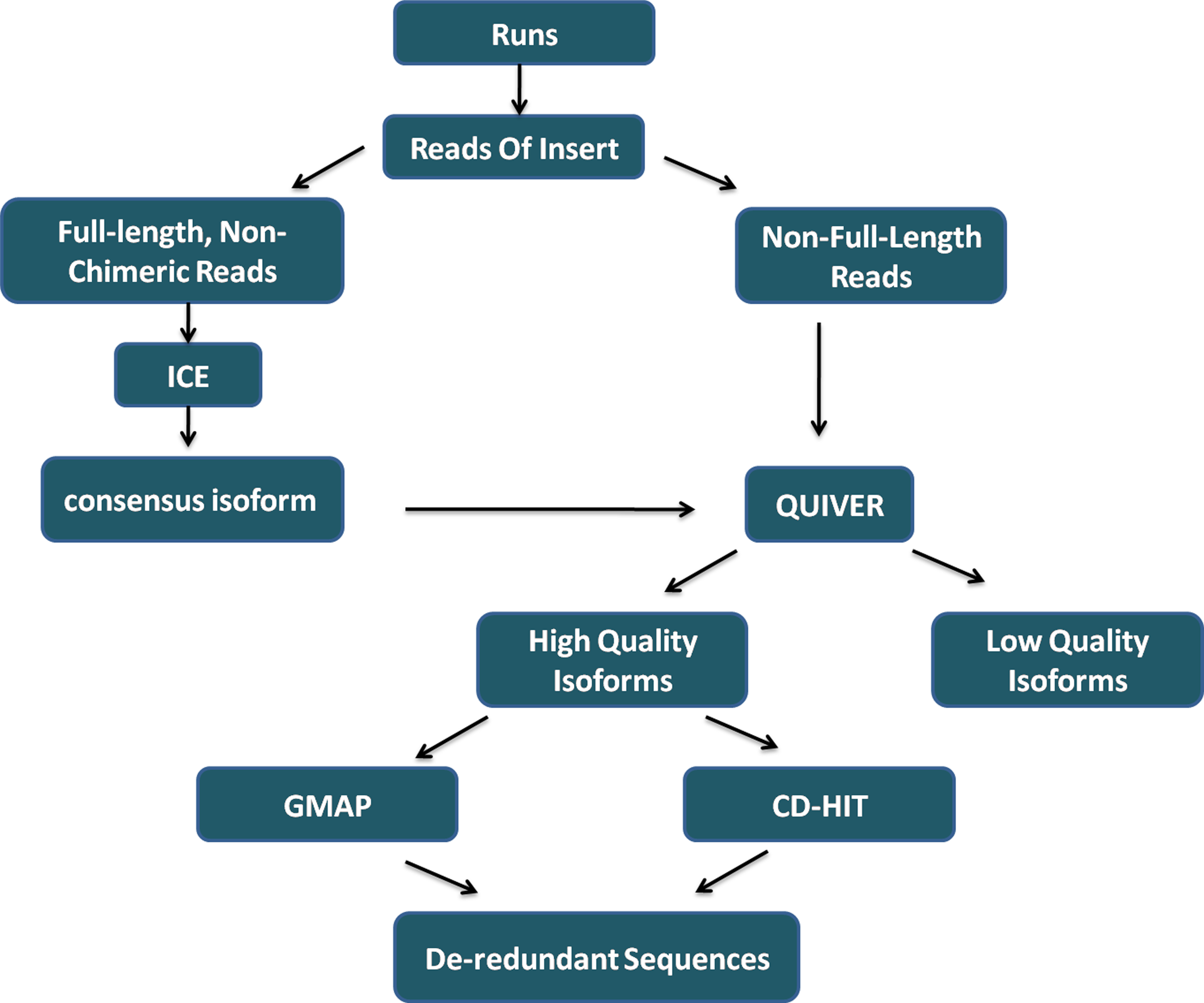

Supplement: Supplementary file 3 — Figure S2. The workflow for data processing in our study. (TIF 655 kb) [file 12864_2018_4946_MOESM3_ESM.tif]

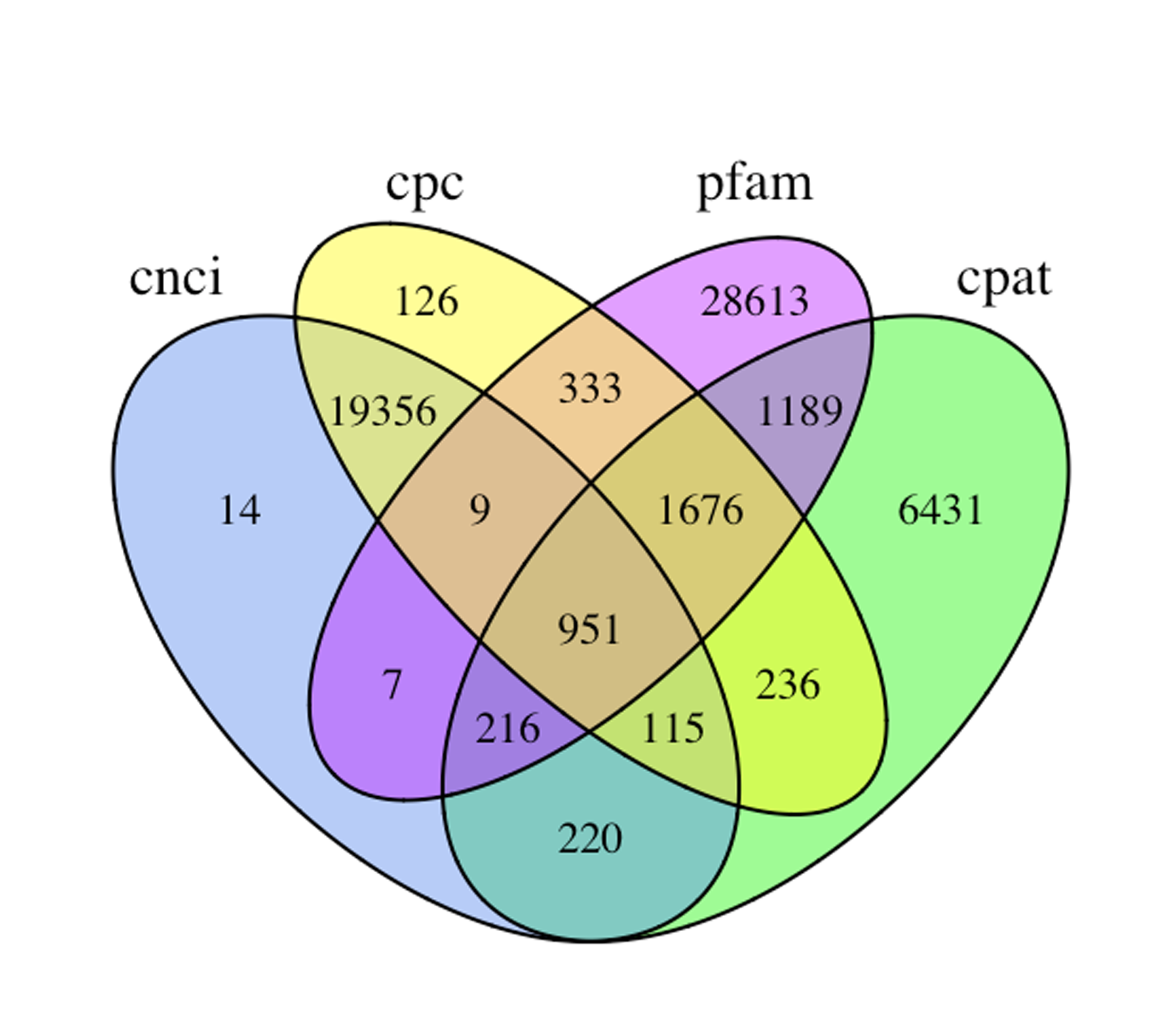

Supplement: Supplementary file 4 — Figure S3. Venn diagram of protein domain prediction. Four types of analysis software (cnci, cpc, pfam and cpat) were used. The intersection of the four software was used for the further lncRNA analyses. (PNG 670 kb) [file 12864_2018_4946_MOESM4_ESM.png]

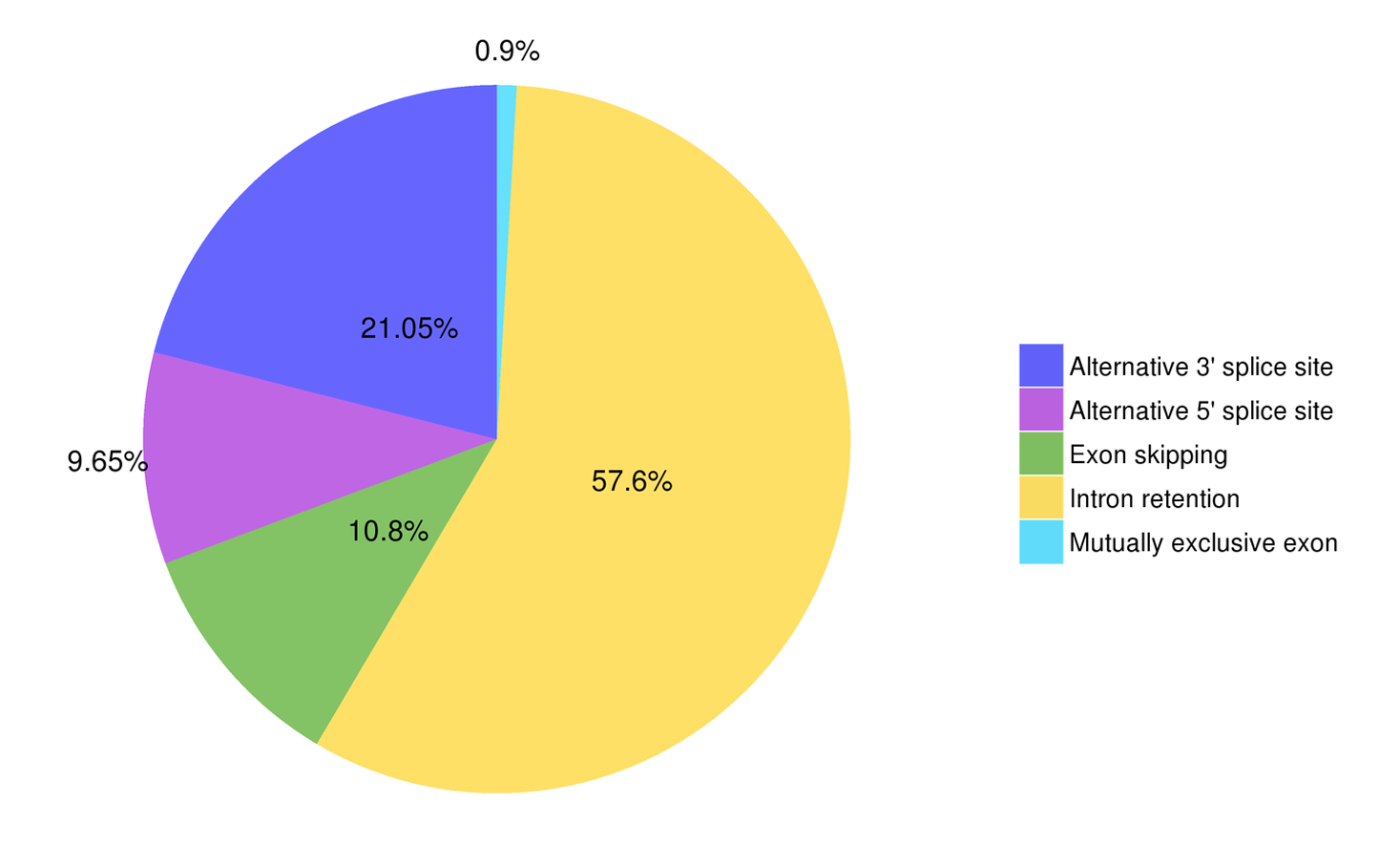

Supplement: Supplementary file 5 — Figure S4. Variable splicing event analyses. The longest transcripts of each gene in the upper genome were extracted as the reference transcript sequence, and cuffcomapare software was used to compare the variable splice sequence of this project with the reference transcript sequence (gff), using ASTALAVISTA software to analyse the variable splicing event. (TIF 231 kb) [file 12864_2018_4946_MOESM5_ESM.tif]

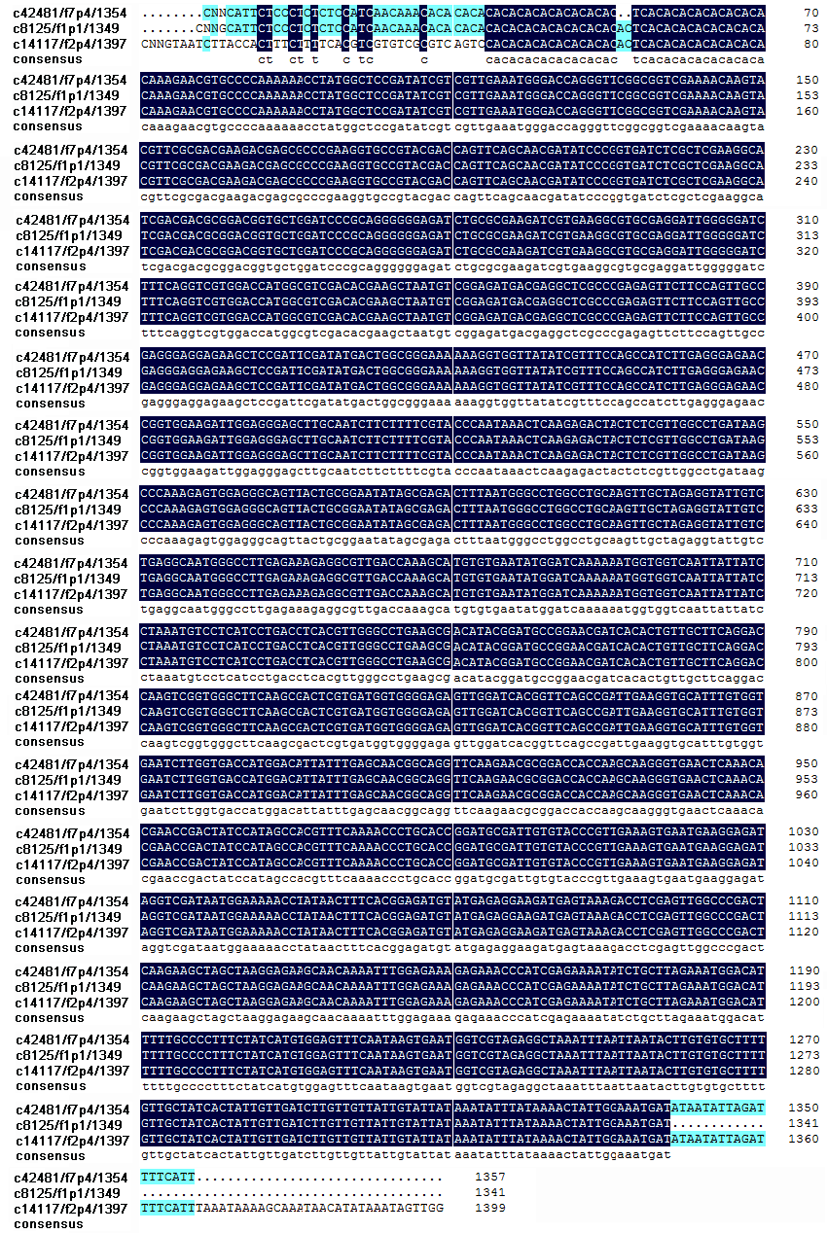

Supplement: Supplementary file 6 — Figure S5. Multiple sequence alignment of F3Hs after the first de-redundancy by CD-HIT. A sequence was considered a sequence with approximately 99% of the sequences. From the results, the three sequences are basically the same, which indicated that the redundancy was not complete. (TIF 1567 kb) [file 12864_2018_4946_MOESM6_ESM.tif]

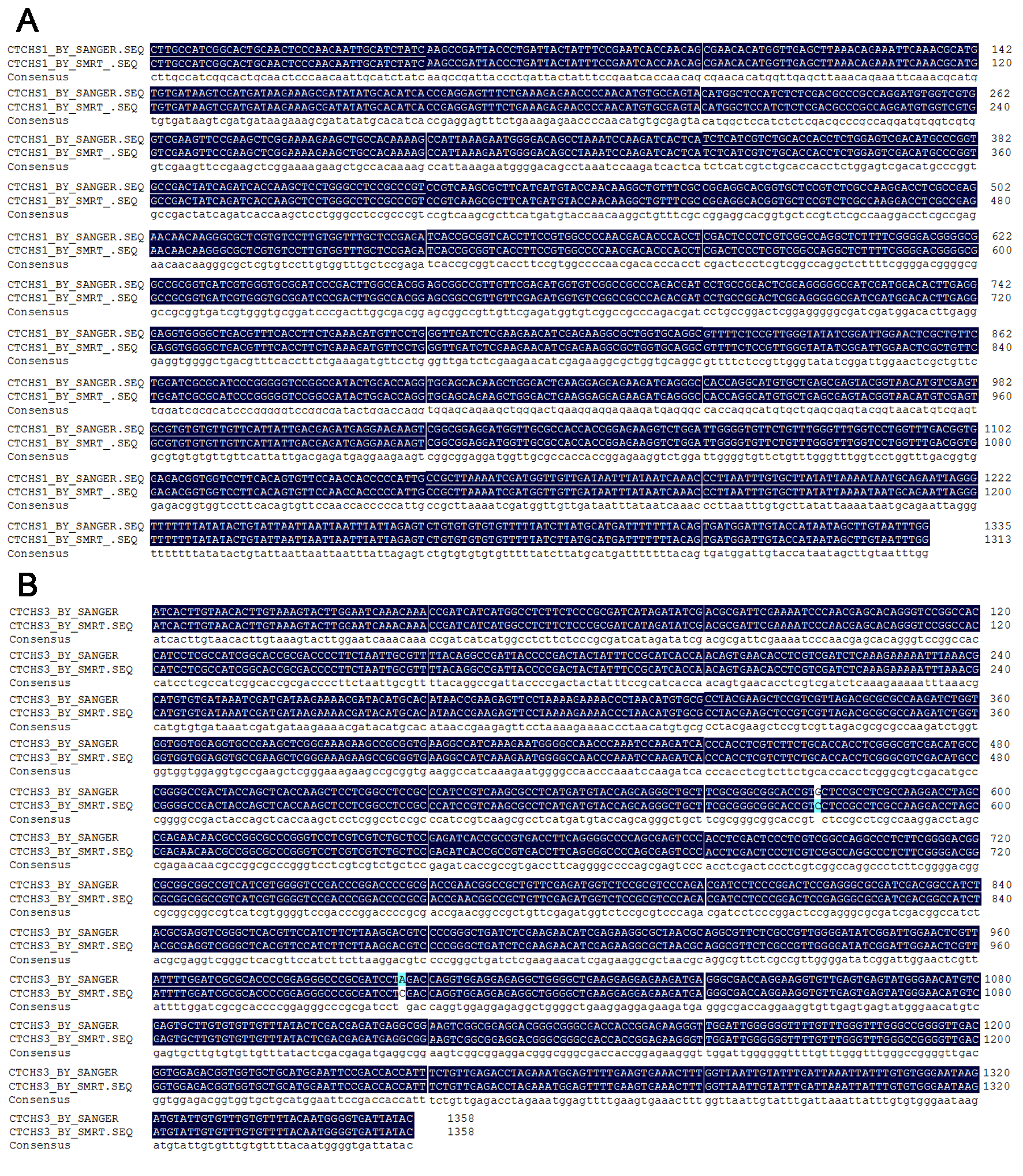

Supplement: Supplementary file 7 — Figure S6. Sequence alignment analysis between the first-generations data and the third-generations data using CtCHS1 and CtCHS3 as the examples. The sequencing results were similar to the third-generations data, with 100% (CtCHS1) and 99% (CtCHS3), respectively (TIF 3997 kb) [file 12864_2018_4946_MOESM7_ESM.tif]

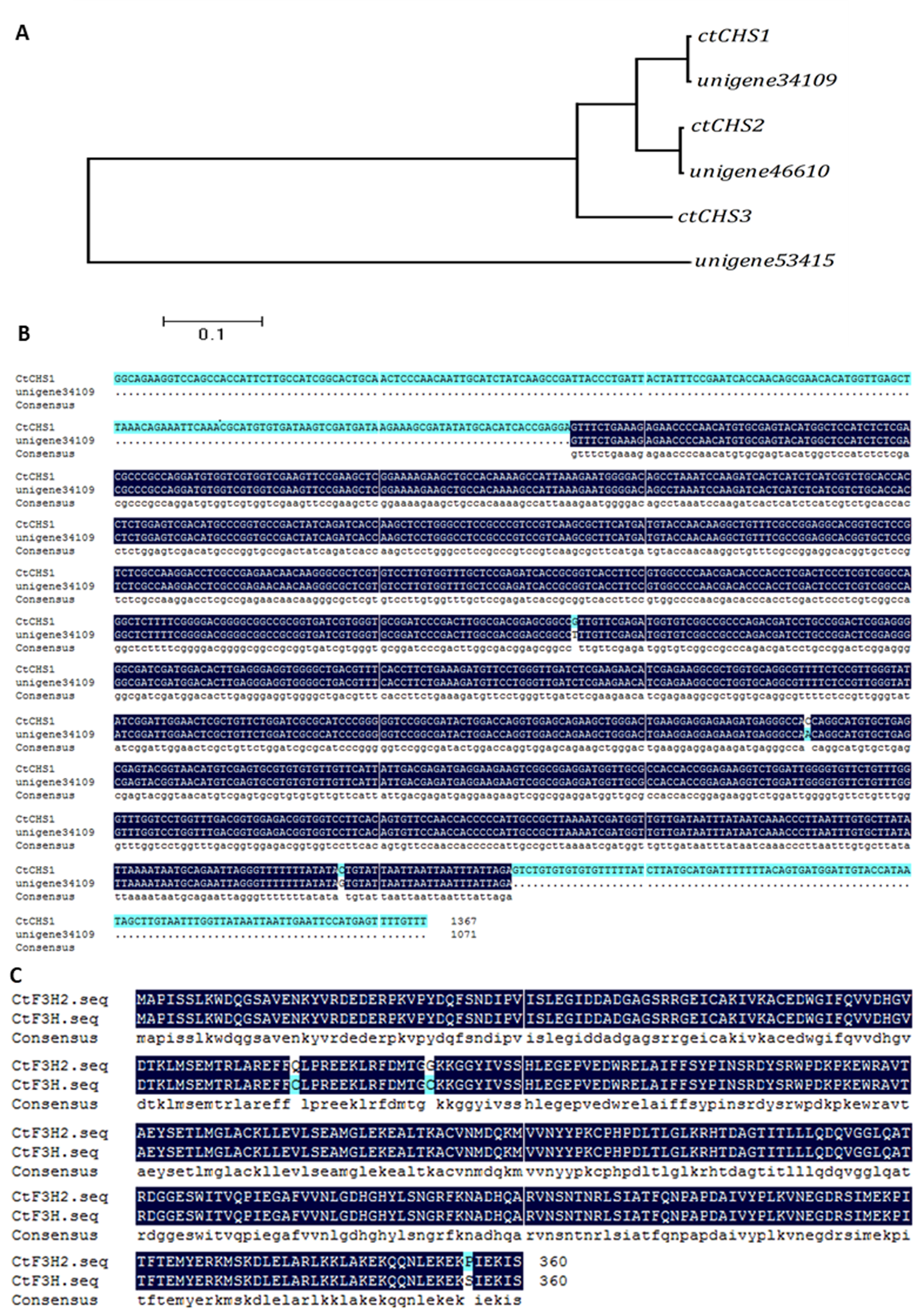

Supplement: Supplementary file 8 — Figure S7. Comparison with reported two generations data. A: The evolutionary analysis of CHS. B: Sequence alignment analysis between CtCHS1 and UNIGENE34109. C: Sequence alignment analysis between CtF3H2 and CtF3H. (TIF 3564 kb) [file 12864_2018_4946_MOESM8_ESM.tif]

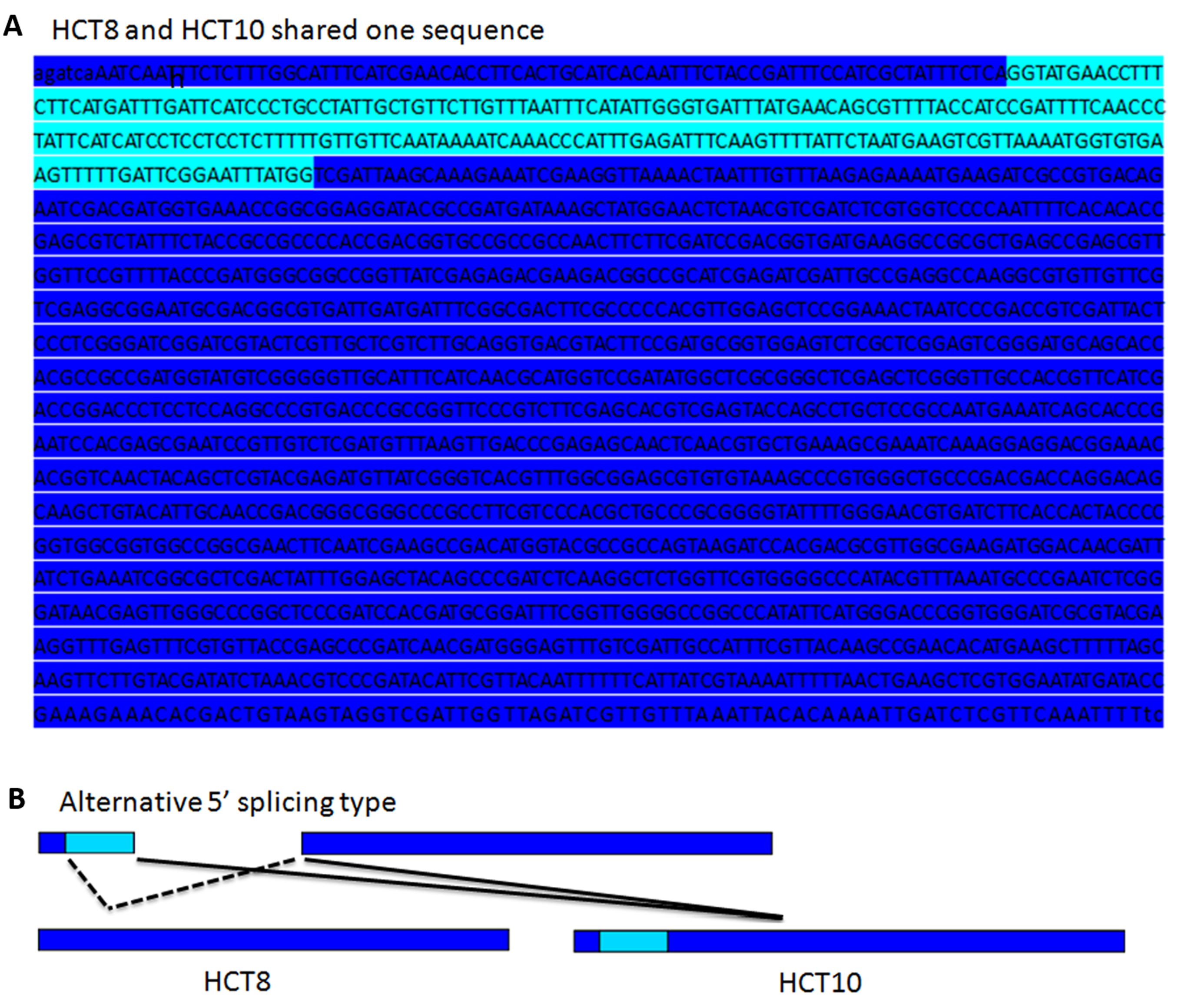

Supplement: Supplementary file 9 — Figure S8. Alternative splice analyses of HCT8 and HCT10. A: Common sequence that HCT8 and HCT10 shared. B: The diagram of the alternative splice of HCT8 and HCT10. (TIFF 2660 kb) [file 12864_2018_4946_MOESM9_ESM.tiff]
